# Cyclic Nonrespiratory Pulse Pressure Oscillations Caused by Atrioventricular Dissociation

**DOI:** 10.1155/2017/7647069

**Published:** 2017-11-29

**Authors:** László Rudas, Péter Hankovszky, András Lovas, Éva Zöllei, Zsolt Molnár

**Affiliations:** Department of Anesthesiology and Intensive Care, Faculty of Medicine, University of Szeged, Szeged, Hungary

## Abstract

Dynamic preload assessment tests, especially pulse pressure variation (PPV) and stroke volume variation (SVV), are increasingly acknowledged in mechanically ventilated patients as being predictors of fluid responsiveness. However, the limitations of this method are often neglected or overlooked. One of the prerequisites for PPV and SVV evaluation, in addition to intermittent positive pressure ventilation, is a “regular heart rhythm,” which may be an ambiguous term. We present a case where, despite a regular (paced) rhythm, atrioventricular dissociation was present and resulted in marked PPV elevation, which subsequently disappeared once sinus rhythm returned. Our case indicates that PPV and SVV should be interpreted with caution when atrioventricular dissociation is present.

## 1. Introduction

Traditional, “static” hemodynamic parameters, such as central venous and pulmonary artery occlusion pressures, have limited capacity for predicting cardiac output changes in response to volume administration. In contrast, the so-called dynamic parameters describe the dynamic interactions of hemodynamic variables in response to a defined perturbation, and the net response indicates the probable circulatory response to fluid therapy [1]. Based on the magnitude of the positive pressure ventilation-induced pulse pressure variation (PPV) or stroke volume variation (SVV), “volume responsiveness” can be defined, which predicts a substantial elevation in cardiac output in response to a fluid bolus [2]. One of the prerequisites of PPV/SVV evaluation is a “regular heart rhythm.” However, this definition does not describe a universally accepted term. We hereby present a case where atrioventricular block necessitated ventricular pacing, and, in spite of the seemingly regular rhythm, the atrioventricular dissociation resulted in “nonrespiratory” PPV augmentation.

## 2. Case Report

A 70-year-old male with a long history of coronary artery disease was brought to our cardiac catheterization laboratory during the night where an acute inferior myocardial infarction was diagnosed. A critical stenosis on the proximal segment of the right coronary artery was successfully stented. However, third-degree atrioventricular (AV) block developed during the procedure necessitating in brief cardiopulmonary resuscitation and the insertion of a temporary ventricular pacemaker (VVI pacing mode, set frequency 80/min). He had to be anaesthetized, intubated, and mechanically ventilated during the catheter intervention, after which he was transferred to our intensive care unit (ICU).

**Figure 1:**
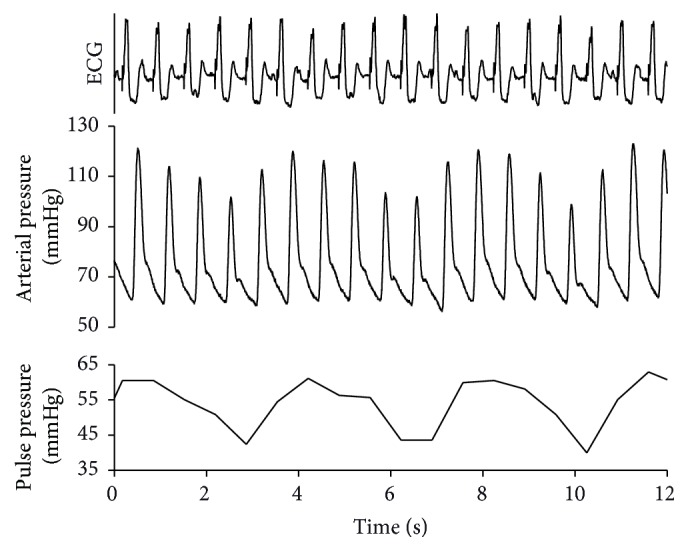
ECG, arterial pressure, and pulse pressure during pacemaker rhythm. The recording was taken shortly after the patient's arrival to the ICU. Pacemaker spikes can be seen before every heartbeat, and the arterial pressure and pulse pressure indicate substantial fluctuations. The recordings were analyzed offline, and a pulse pressure diagram was also added.

On admission, he was hemodynamically unstable with marked arterial pressure fluctuation noted on the first ECG and arterial pressure curve recordings (Figure 1). Initially, his hypotension was treated with norepinephrine 0.5 *μ*g/kg/min, which had to be increased up to 1.2 *μ*g/kg/min to maintain an acceptable mean arterial pressure. Arterial blood gases showed severe metabolic acidosis with pH of 7.26, lactate of 4.5 mmol/l, HCO_3_ of 13.4 mmol/l, and a base deficit of 13.6 mmol/l. Therefore, invasive hemodynamic monitoring was commenced with transpulmonary thermodilution and pulse contour analysis (PiCCO, PULSION, Germany). The first measurements revealed a low cardiac index (CI) of 2.2 l/min/m^2^. Based on the corresponding hemodynamic indices (SVI: 26 ml/m^2^, *dP*/*dt*_max_: 1545, and SVRI: 2230 dyn*∗*s*∗*cm^−5^), dobutamine dose, 3.0 *μ*g/kg/min, was added to the therapy. His spontaneous and paced rhythm alternated.

**Figure 2:**
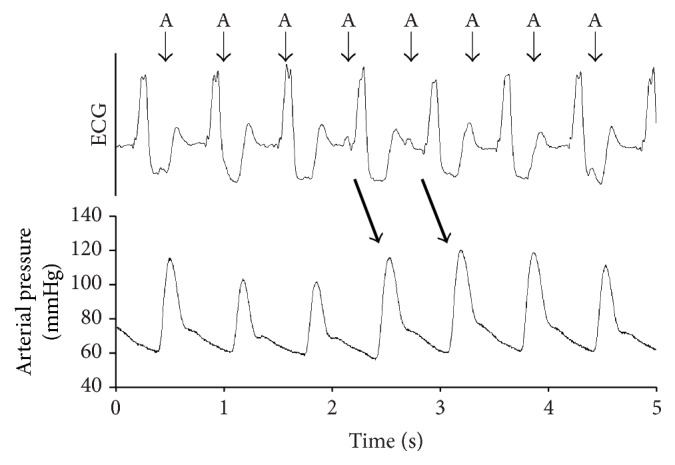
Relationship of sinus and paced cardiac activity and its effect on arterial pressure. The ECG strip shows a paced ventricular “regular” rhythm, where atrioventricular dissociation is still present. The small arrows indicate atrial activity (“A”). The coincidence of normal AV sequence results in substantial arterial pressure augmentation (indicated by the thick arrows). For more details, read the text.

A more detailed evaluation of the ECG and blood pressure recordings revealed an obvious interference between the paced ventricular and spontaneous atrial (sinus) activities (Figure 2). When this interference resulted in a normal AV sequence, there was a substantial increase in the area under the arterial pressure curve, indicating an augmented stroke volume.

**Figure 3:**
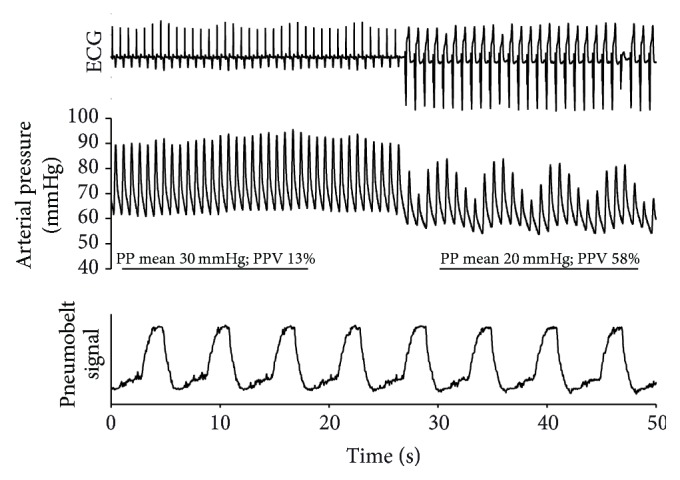
Change of pulse pressure variation (PPV) during sinus and pacemaker rhythm. ECG, arterial pressure, and noncalibrated pneumobelt signals were recorded during the dynamic preload test. While the initial segment of the arterial pressure panel during sinus rhythm indicates minimal changes in pulse pressure (PP) resulting in borderline PPV, the switch to ventricular pacemaker rhythm is accompanied by enormous pressure fluctuations and a severalfold increase in PPV. The straight lines indicate the 3 breathing cycles detected by the pneumobelt during the first and second part of recording. Based on the pneumobelt signal, the arterial pressure swings occurring during the pacemaker rhythm seem to be unrelated to the ventilation.

By the second day, his heart rate seemed regular, and intermittent restoration of the normal AV conduction was noted in addition to the paced rhythm. At this time, the patient was mechanically ventilated in pressure control mode with 10/min breathing frequency with no spontaneous breathing efforts as he was kept under deep sedation. Therefore, this scenario (regular rhythm, controlled mechanical ventilation) seemed ideal to assess fluid responsiveness using PPV. Furthermore, the alternative sinus and VVI-paced periods allowed comparison of the hemodynamic responses during these two different rhythms (Figure 3). For the brief period of the test, the tidal volume was increased to 9 ml/kg. In order to be able to record changes in cardiac and respiratory mechanics on the same recording, we applied a device called the pneumobelt, a silicone tube hooked around the abdomen, which measures pressure changes in response to mechanical ventilation. A switch from sinus rhythm to VVI pacemaker rhythm resulted in general hemodynamic deterioration. In addition to the drop in mean pulse pressure from an already low value of 30 mmHg to an even lower value of 20 mmHg, this change was accompanied by a large increase in PPV, from 13% to 58% (Figure 3). The 13% PPV during sinus rhythm was just around the cut-off range for volume responsiveness. However, the >4-fold increase in PPV when the VVI pacemaker took over by definition would normally suggest volume responsiveness. However, we decided not to give fluid, taking into consideration the full clinical picture, and we carried on treating him for cardiogenic shock.

The patient's subsequent course was uneventful. The sinus rhythm stabilized so that he could be successfully weaned from the ventilator within 24 hours. The catecholamine support was also terminated by the third day of observation and eventually the pacemaker could be removed. Echocardiography revealed satisfactory global left ventricular function (EF 54%), with inferobasal akinesis. No significant mitral insufficiency was seen. He was discharged to the cardiology ward for further treatment.

## 3. Discussion

Dynamic preload assessment with PPV or SVV in mechanically ventilated patients is increasingly acknowledged in our daily routine but is still underused in clinical practice [3]. Despite the fact that these are considered to be the most sensitive and specific indicators of fluid responsiveness, there are several prerequisites for appropriate assessment and there are several circumstances that can limit the interpretation of the results (Table 1) [4–8]. Unfortunately, not all of these components are obvious or known by many anesthesiologists and intensivists [7]. “Regular rhythm” may seem like a simple requirement, but, surprisingly, the definition is ambiguous in various papers. Certain authors specify sinus rhythm [4, 7, 8] and others simply define regular rhythm as “lack of irregularity” [9] (at least for the duration of a single respiratory cycle [10]) and others talk about the absence of arrhythmia [5, 6]. These terms are confusing. Normal sinus rhythm, especially in healthy young subjects, can be quite irregular, and on the other hand certain forms of arrhythmias are actually regular.

In this current case, the patient had regular (paced) rhythm but the atrioventricular dissociation resulted in a “nonrespiratory” PPV augmentation. This response, however, is not “false” in the sense that it is related to true preload disturbances caused not by changes in the circulating blood volume but rather by the spontaneous atrial activity. Cyclic synchrony and dyssynchrony of the atrial “booster pump” resulted in varying ventricular filling ranging from optimal to very abnormal. The latter was the case when the atria and the ventricles contracted simultaneously, resulting in abnormally high atrial pressure waves, which actually impeded venous return.

One of the main clues in our case was the simultaneous assessment of the ECG trace and arterial pressure curves, which put in context the noninvasively measured breathing pattern detected by the pneumobelt. This revealed an asynchrony between the breathing cycle and the observed major changes in PPV. This information provided by the pneumobelt was only applied because we wanted real-time comprehensive documentation. However, this could easily be replaced by the observation of the ventilator monitor.

It is important to note that these observations do not necessarily mean that pacemaker rhythm always excludes the possibility of PPV assessment. In patients, for example, with complete AV block and VVI pacing in the setting of permanent atrial fibrillation or atrial standstill, a dynamic preload test still could be an informative procedure. We have learnt from cardiac resynchronization studies that modulation of AV delay has a tremendous impact on the pulse amplitude [11]. Cyclic changes in AV delay, as seen in our case, contaminate the PPV pattern.

## 4. Conclusion

Definition of a “regular rhythm,” a prerequisite for dynamic preload tests in mechanically ventilated patients, is ambiguous. Our case shows that mechanistic implication of PPV as an indicator of fluid responsiveness when the heart rate is considered regular could have led to incorrect conclusions. However, this was overcome by thorough simultaneous observation of the ECG, arterial pressure curves, and the breathing cycle. We also feel that clarification of the current exclusion criteria may be warranted.

## Figures and Tables

**Table 1:** Prerequisites for dynamic preload assessment using ventilation-induced hemodynamic alterations.

(i) Tidal volume > 8 ml/kg
(ii) Heart rate/respiratory rate ratio > 3.6
(iii) Closed chest
(iv) No abdominal hypertension
(v) No spontaneous respiratory efforts
(vi) No right ventricular failure
(vii) Regular rhythm
